# Influence of bethanechol on salivary parameters in irradiated patients

**DOI:** 10.4317/medoral.21395

**Published:** 2016-12-06

**Authors:** Claudia Cotomacio, Luana Campos, Alyne Simões, Graziela Jaguar, Edgard-Michel Crosato, Fabio Alves

**Affiliations:** 1 Stomatology Department - School of Dentistry, University of São Paulo, São Paulo, Brazil; 2 Oral Biology Research Center, Department of Biomaterials and Oral Biochemistry - School of Dentistry, University of São Paulo, São Paulo, Brazil; 3 Stomatology Department - A.C. Camargo Cancer Center, São Paulo, Brazil; 4 Social Dentistry Department - School of Dentistry, University of São Paulo, São Paulo, Brazil

## Abstract

**Background:**

Some studies have shown evidence that the prophylactic use of bethanechol chloride (BC) may be useful in preventing the incidence and/or severity of xerostomia (XT). However, the indication of BC in irradiated patients with XT needs to be better characterized. The study aimed to evaluate the influence of BC on XT, salivary flow rate, and salivary composition in patients previously submitted to head and neck radiotherapy.

**Material and Methods:**

Forty five irradiated patients complaining of XT used 50 mg/day of BC for 3 months, and the salivary parameters were evaluated in 4 Phases (Before BC therapy, after one month of BC, 2 months of BC, and 3 months of BC). Biochemical analysis included buffering capacity; pH; total protein concentration (TP); amylase concentration (AM); catalase (CAT) and peroxidase (PX) activities. In addition, unstimulated and stimulated salivary flow rates were determined and XT was classified.

**Results:**

According to the XT grading system used, patients showed improvement in XT between Phase 1, and Phases 2, 3 and 4. In addition, some changes were observed in TP concentration (decreased); AM concentration (increased); and PX and CAT activities (decreased and increased, respectively) after Phase 2, for stimulated saliva collection (*p*<0.05).

**Conclusions:**

Our results suggested that when BC was used to treat salivary gland dysfunction induced by head and neck radiotherapy, improvement in XT symptoms, and some changes in saliva composition were shown.

**Key words:**Radiotherapy, xerostomia, hyposalivation, saliva, biochemistry.

## Introduction

The radiotherapy (RT) is indicated as primary, adjuvant to surgery, or palliative therapy for tumors in the head and neck region (1). However, the secondary effects of the RT are a challenge to the professionals involved in the management of these patients. Oral mucositis; taste alterations; trismus; dental caries; progressive periodontal disease; osteoradionecrosis; hyposalivation and xerostomia (XT) are the main disturbing side effects related to head and neck RT (2).

XT may cause an important impact on patients’ quality of life, mainly because it may last throughout their entire lifetime (3). Xerostomic patients usually present difficulties with chewing, swallowing, tasting or speaking (4).

The mechanisms by which RT causes damage to the salivary glands have not yet been completely elucidated. Konings *et al.* (5) proposed that RT promotes injuries to the signal transduction system of cell membranes in salivary glands. Free radicals and peroxides bind to acinar cell membrane receptors, blocking activation of the entire intracellular protein signaling cascade. Thus, XT and hyposalivation are caused by impaired saliva production. Some authors (6,7), have suggested that the salivary flow is preserved when the doses of RT are between 26 Gy and 32 Gy; and Jensen *et al.* (8) related that a dose higher than 52 Gy causes a permanent damage to salivary glands.

Bethanechol Chloride (BC), a cholinergic agonist, has been considered an option to increase the salivary flow rate during head and neck RT, because it has fewer side effects than pilocarpine (9,10). Recently, our group, in a double-blind study, demonstrated that the prophylactic use of BC improved the salivary flow rate, XT symptoms and quality of life of patients submitted to radiotherapy (2). However, few studies have demonstrated the benefits of BC for treating salivary gland hypofunction in patients previously submitted radiotherapy in the head and neck regions. Thus, the aim of this study was to evaluate the effect of BC therapy on XT, hyposalivation and saliva composition in head and neck irradiated patients.

## Material and Methods

This interventional study was conducted at the University of São Paulo from March 2013 to January 2015. The ethics committee approved this study (Nº 166.104) and written informed consent was obtained from all patients. All procedures performed in this study, involving human participants, were in accordance with the ethical standards of the institutional and national research committee and with the Helsinki Declaration of 1975 as revised in 1983.

- Patients

This interventional study enrolled 45 patients of the “Liga Interdisciplinar de Neoplasias Bucais” (LINB) of the University of São Paulo, who were submitted to head and neck RT with a minimal dose of 60Gys; and complained of xerostomia. All the patients were assessed for eligibility. The grade of XT was evaluated monthly, from baseline to 3 months after treatment, using Eisbruch’ scale (11). Exclusion criteria were: patients with hypersensitivity to BC; hypotension; hyperthyroidism; peptic ulcer disease; epilepsy; angina; those with parkinsonism; smoking; patients using tricyclic antidepressant and antihistamine drugs.

- Study design 

This study was designed to test BC therapy after head and neck RT, therefore, the follow up time was 3 months, divided into four Phases: before BC administration (Phase 1) and after 1 (Phase 2), 2 (Phase 3) and 3 (Phase 4) months. Phases 2, 3 and 4 were compared with Phase 1 in all the statistical tests. According to the ethical principles Phase 1 (assessments before BC administration) was used as a control parameter for the subsequent Phases (after BC administration). The 3 months follow-up was chosen according to the evidences and effectiveness of BC treatment found in a similar study (2) and prophylactic treatment with agonists drugs, such as pilocarpine and bethanechol, have shown a positive effect on salivary flow in animals and humans within 30 days after radiotherapy (2,12).

- Bethanechol protocol: BC therapy consisted of one tablet twice a day for 3 months (50mg/day). Bethanechol toxicity was scored using National Cancer Institute Common Terminology Criteria for Adverse Events - NCI CTCAE, v3.0 (2,13). This questionnaire was designed to address specific acute and late symptom-related toxicity experienced by cancer patients. Once a month, the questionnaire was applied to all patients, to control possible bethanechol-related symptoms.

- Xerostomia: The grade of XT was assessed by a single professional, monthly from Phase 1 to Phase 4 using a grading system, according to the subjective measures of the Eisbruch *et al.* (11) (2003) scale as follows.

Grade 1: mild dryness with no disability.

Grade 2: dryness requiring additional fluids for swallowing.

Grade 3: dryness causing dietary alterations, interference with sleep, speaking or other activities.

To compute the correlation between XT and the Phases, mild XT was considered Grade 1 and severe XT, Grade 2 or 3.

- Saliva collection

Sialometry was evaluated by unstimulated whole saliva (UWS) and stimulated whole saliva (SWS). Saliva was collected in all the experimental time intervals; always in the morning, between 8h30 a.m. and 11h30 a.m., to minimize the effects of the circadian rhythm in salivary glands. All patients were instructed not to eat, drink or smoke 1 hour before saliva collection. In all types of collections, the patients were placed in a comfortably seated position and asked to swallow any saliva in the mouth, immediately before the tests. For the UWS, the patient started spitting the spontaneously produced saliva into a plastic funnel connected to a graduated tube, for 10 minutes. The last collection was SWS, using one drop of solution of citric acid on the dorsum of the tongue every 30 seconds for 10 minutes, then the patients were instructed to spit the saliva into another tube (14). After collection, the samples were weighed and the salivary flow rate was calculated and adjusted in ml/min (15). For samples in which a sufficient volume of saliva was acquired (> 0.5 ml) the pH and the Buffer Capacity evaluations were performed immediately after the UWS collections. (These tests could not be performed for SWS, due to the interference of citric acid.) After this, all the saliva samples were kept on crushed ice, and taken to the Oral Biology Laboratory of the University of São Paulo (USP), were the samples were centrifuged and then stored at -80ºC until biochemical analyses were performed (14).

- Biochemical analyses 

pH and Buffer Capacity: To evaluate buffer capacity and pH, 0.5 mL of UWS samples were used to perform the pH analysis by means of a Digimed 2D portable pH meter, immediately after the saliva collections. After this, the buffer capacity was evaluated by titration of the sample with a HCl 0.01 N solution. Before measuring began, the pH meter was calibrated with standard pH solutions at the values 7.0 and 4.0. The buffer capacity was expressed by the quantity of HCl required to decrease the sample pH to 4.0.

Total protein concentration (TP): The TP in saliva was determined by the Lowry *et al.*(16) (1951) method, using bovine albumin as standard. The readouts were made at 660 nm by using an ELISA plate reader.

Amylase (AM): This was determined by Bellavia *et al.* (17) (1979) . The readouts were made at 530nm in a Beckman DU-68 spectrophotometer.

Peroxidase (PX) and Catalase activity (CAT): PX was determined according to Anderson (18) (1986) and Chandra *et al.* (19) (1977) (using a lactoperoxidase solution as standard, and the absorbance was measured at 460 nm. The CAT activity was measured at 240nm in a Beckman DU-68 spectrophotometer, according to the methods of Aebi (20) (1984) . The difference in absorbance per unit time was the measure of the catalase activity.

- Statistical Analyses

Statistical analyses were performed using Minitab 16.1 and MedCalc 15.2.2 software packages. The McNemar test was applied to compare results of XT grades in each Phase. After this, Biochemical and Sialometry data were submitted to analysis of variance followed by 2 sample T tests. All the results were obtained with a confidence interval of 95%.

## Results

A total of 45 patients were assessed and all completed Phase 1; 41 completed Phase 2 (2 were excluded due to hypotension - a side effect of the BC; and 2 did not keep the appointment); 37 completed Phase 3 (4 did not keep the appointment); and 30 completed Phase 4 (a total of 7 patients were excluded; 2 due to thyroid alteration; 1 died; 2 due to recurrence of the tumor; and 2 did not keep the appointment). A total of 15 patients did not complete all Phases of the study (a side effect of BC was considered in only 2 patients) ( Table 1).

**Table 1:**
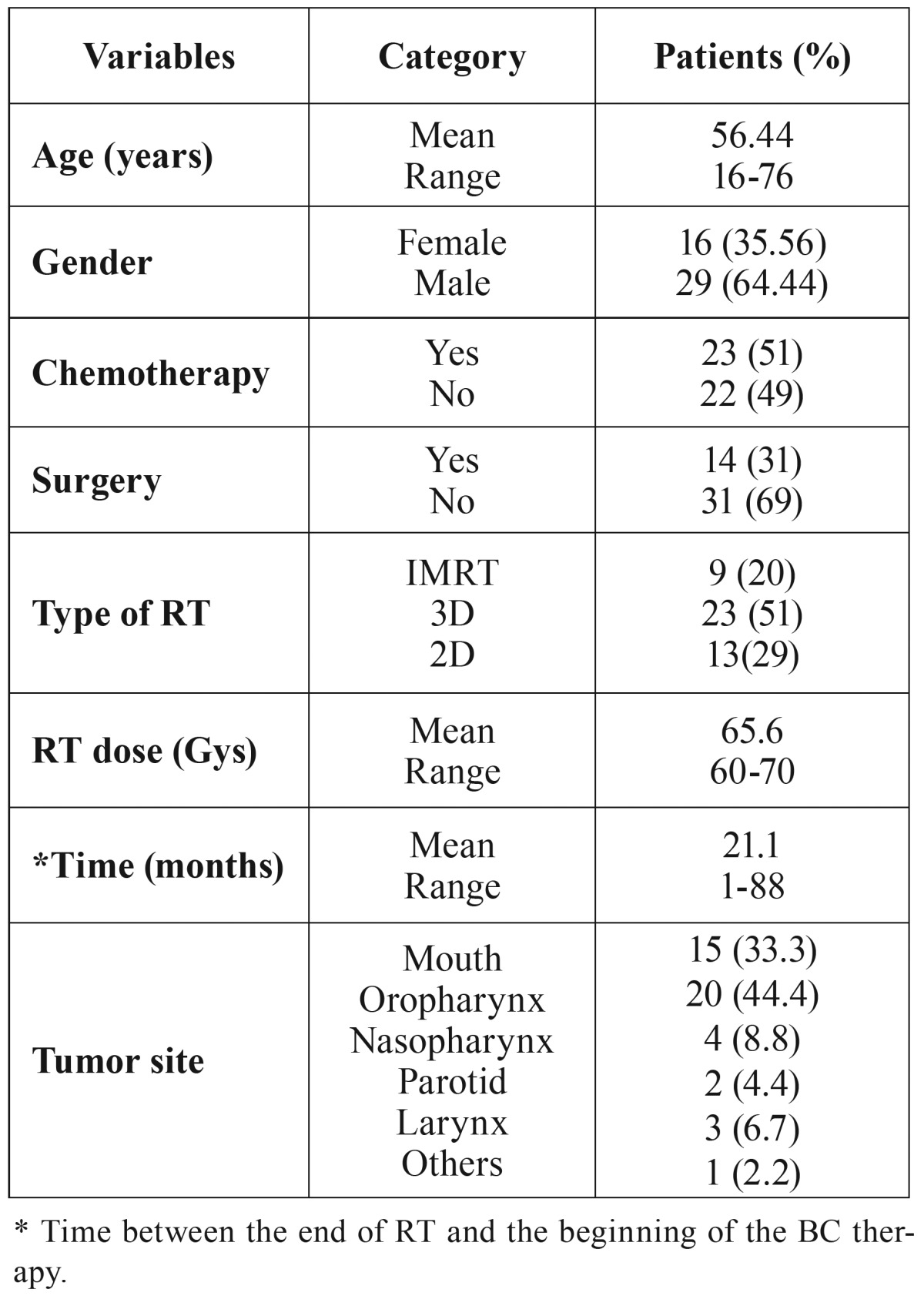
Clinical features of the 45 patients.

**Figure 1:**
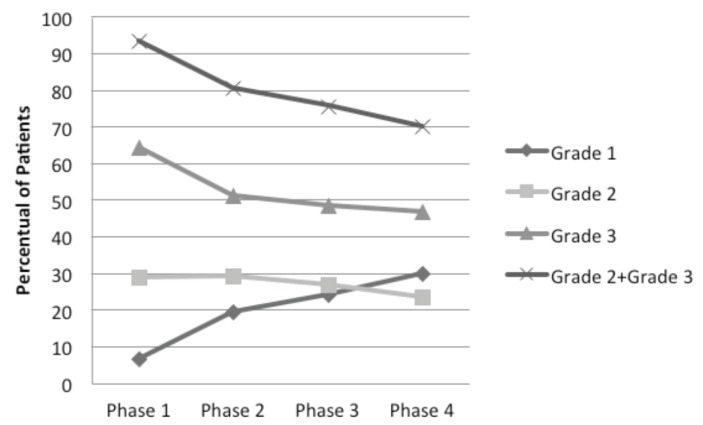
Distribution of the patients according to xerostomia grades in all phases.(Microsoft Excel 2007).

- Xerostomia evaluation:

During Phase 1 (before BC therapy), most of the patients had severe XT (93.3%); only 3 patients (6.7%) presented XT Grade 1. There was a gradual improvement in the grade of XT during the next 3 Phases of the study. In Phases 2, 3 and 4, the percentage of severe XT decreased to 80.5%, 75.7% to 70%, respectively. Inversely, mild XT increased from 19.5% (Phase 1), to 24.3% (Phase 2) and to 30% (Phase 3) (Fig. 1,  Table 2) 

**Table 2:**
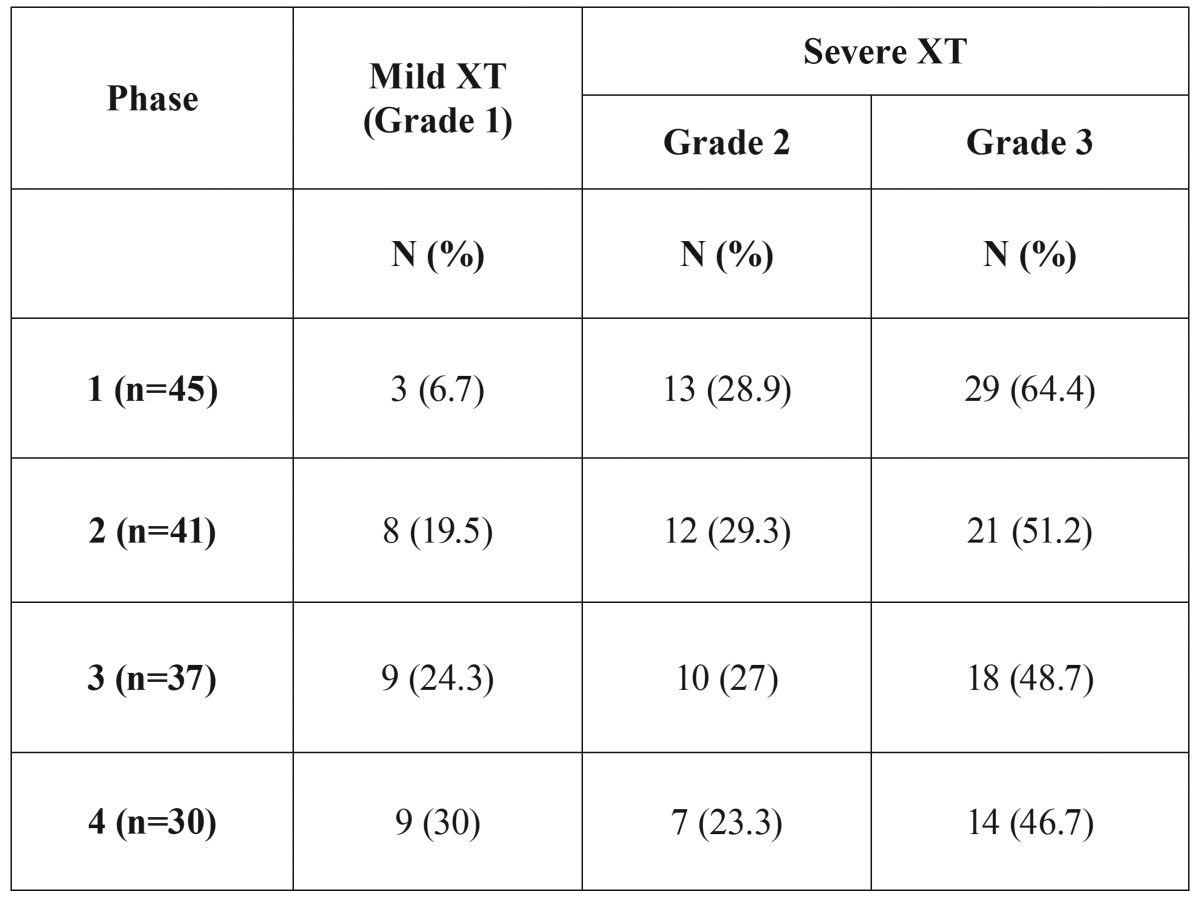
XT Grades (Eisbruch *et al.* 2003) distributed in each Phase of the study.

When mild XT was compared with severe XT, there was significant improvement between Phase 1 and others ( Table 3).

**Table 3:**
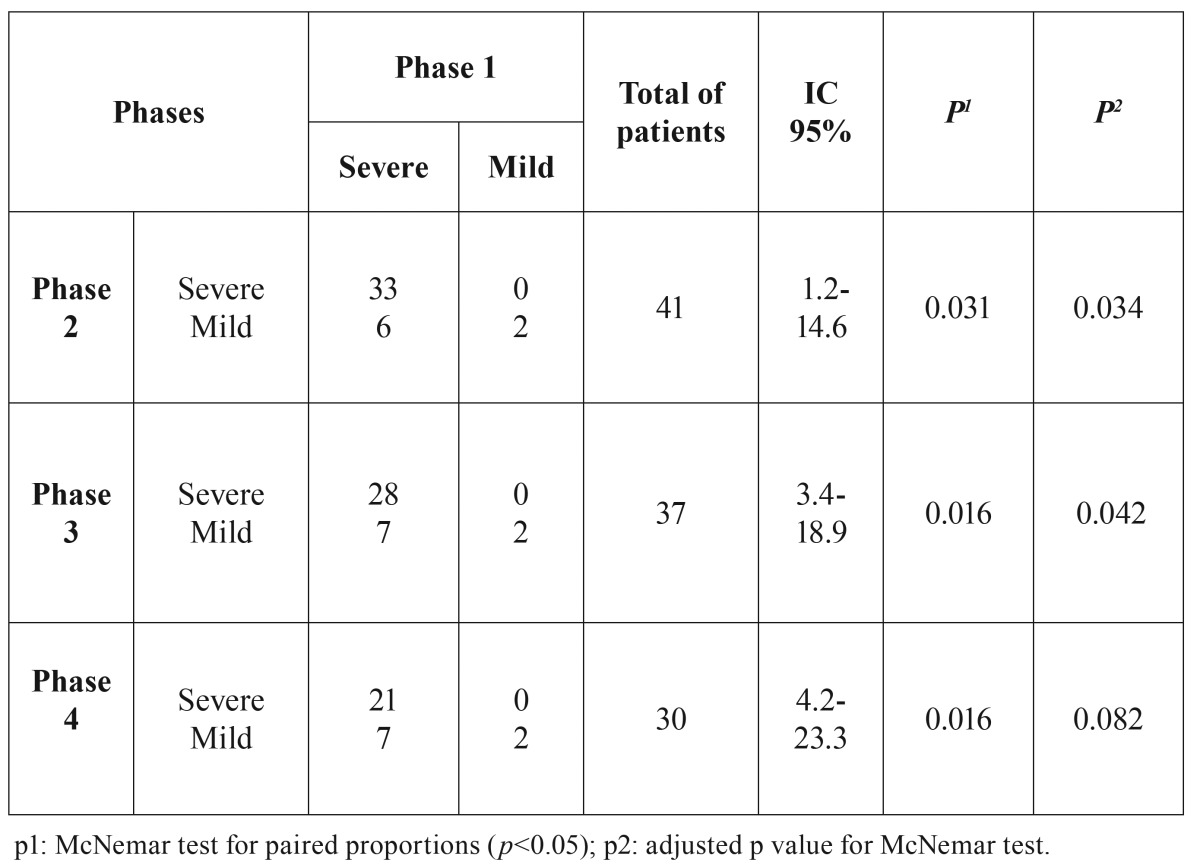
Contingency table for the phase 1 XT grades (Eisbruch *et al.* 2003) compared to the other phases (2, 3 and 4). For this association, the phase 1 was paired to each phase.

- Sialometry evaluation:

The sialometry results, for both UWS and SWS, showed no significant differences in the experimental time intervals (Phase 1 to 4) ( Table 4).

**Table 4:**
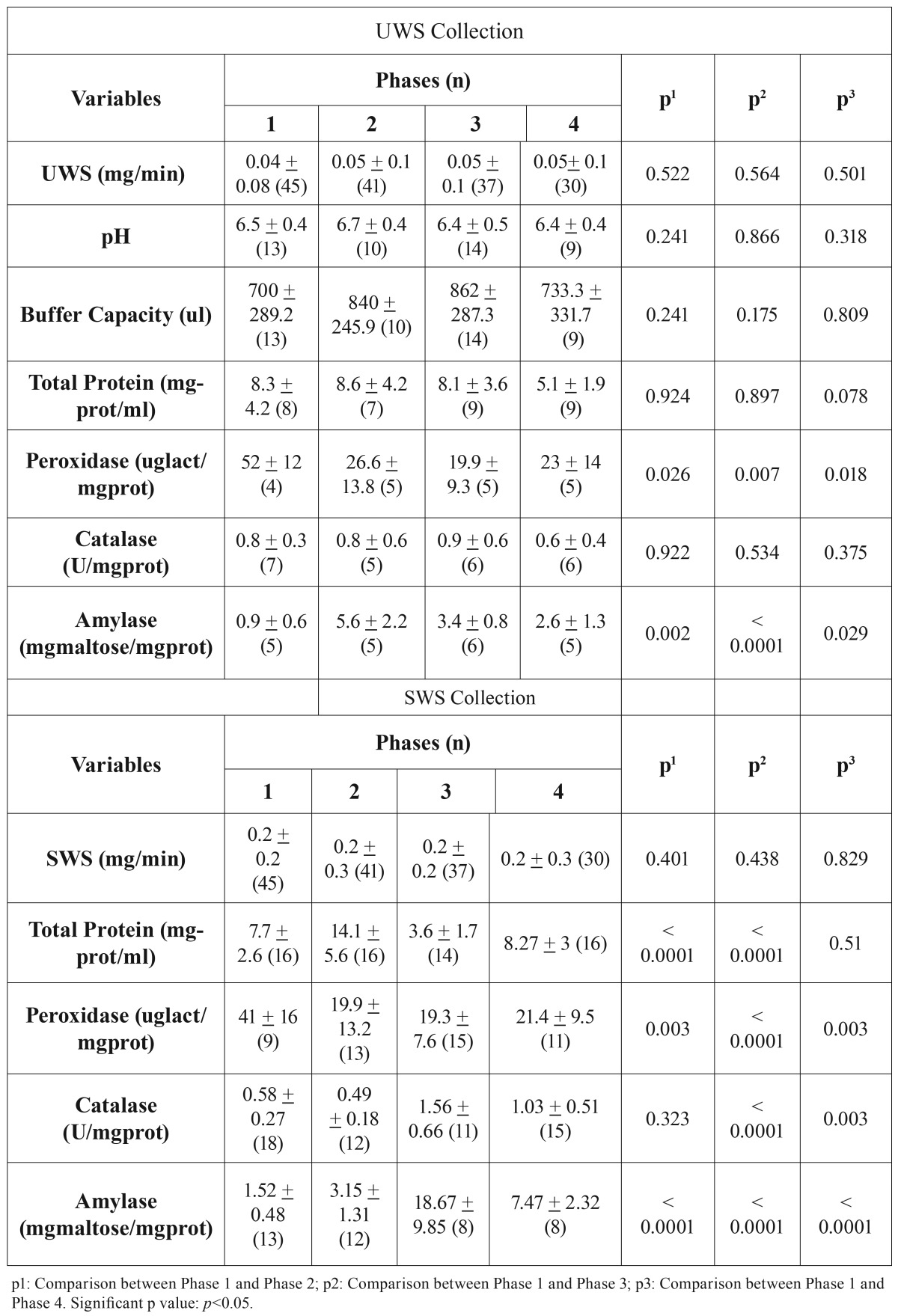
Mean of salivary flow unstimulated whole saliva (UWS) and stimulated whole saliva (SWS). pH and Buffer Capacity (for UWS), Total Protein, Peroxidase, Catalase and Amylase, compared in each phase of the study.

- Biochemical analyses:

Buffer capacity and pH values showed no significant difference between the Phases ( Table 4). However, saliva composition (both UWS and SWS) showed the following differences:

Total Protein: In UWS, the TP test showed no difference between the Phases ( Table 4). Whereas for SWS collection, TP fluctuated: an increase between Phases 1 and 2 (*p*<0.0001); decrease between Phases 1 and 3 (*p*<0.0001); Phase 4, showed a return to values similar to those in Phase 1 (*p*=0.51) ( Table 4).

Peroxidase: In UWS collection, there was a decrease in PX activity by comparing Phase 1 with Phase 2 (*p*=0.026); 3 (*p*=0.007) and 4 (*p*=0.018) ( Table 4). For SWS, there also was a decrease in this enzyme activity, by comparing Phase 1 with the other Phases; 2 (*p*=0.003); 3(*p*=<0.0001); and 4 (*p*=0.003) ( Table 4).

Catalase: In UWS, there was no significant difference between the study Phases (*p*<0.05) ( Table 4). However, in SWS, it was found an increase in CT when comparing Phase 1 with Phases 3 (*p*<0.0001) and 4 (*p*=0.003) ( Table 4).

Amylase: There was an increase of AM concentration in UWS collection between the Phases 1 and 2(*p*=0.002); 3 (*p*<0.0001) and 4 (*p*=0.029); ( Table 4). For SWS collection, Phase 1 was compared to the others Phases (*p*<0.0001) ( Table 4).

## Discussion

Recently, our group demonstrated the benefits of BC used as prophylactic therapy to prevent radiotherapy-induced XT, mainly in patients treated with IMRT. However, in some patients their XT scores increased after they stopped taking the drug, therefore the duration of BC administration needs to be better defined (2). In the present study, all patients had been treated with high doses of RT for head and neck cancers, and the action of BC was evaluated. There is no fully effective therapy for the treatment of XT. Artificial saliva, oral lubricants and pilocarpine are commonly used (12,21). Some patients still prefer to drink water constantly because they do not adapt to such therapies. Thus, the use of BC could benefit these previously irradiated patients, with minimal side effects.

Cholinergic agonists have often been studied for the treatment of salivary glands damaged by RT (12). BC has an action similar to that of pilocarpine: both are cholinergic agonists and increase glandular function by stimulating the muscarinic and nicotinic receptors (22). However, as an advantage, BC has fewer side effects than pilocarpine (9,10,23). Epstein *et al.* (9) (1994) showed a significant increase in both UWS (=0.003) and SWS (*p*=0.001) after BC therapy in irradiated patients. Later, Grosky *et al.* (10) (2004) compared BC and pilocarpine in 42 patients with XT after RT and both drugs showed statistically significant increase in SWS (*p*=0.01). Jham *et al.* (23) (2007) found an increase of UWS immediately after RT (*p* = 0.03). In a recent double-blind study, our group showed that the prophylactic use of BC during the RT showed significant efficacy in preventing severe XT (*p*<0.001) and hyposalivation (*p*<0.05) (2). In the present study, we found a substantial reduction in XT Grade 3 (severe) from Phase 1 (64.4%) to Phase 4 (46.7%). However, it was not possible to find significant differences in the salivary flow rate.

RT affects the composition and the quality of saliva, and pH values are known to be reduced to acidic levels after the RT, due to the reduction in bicarbonate levels (24). However, in this study, BC therapy was unable to improve the buffering capacity and pH of saliva in these patients. Hannig *et al.* (25) (2006) reported that the RT may influence the salivary protein concentrations, mainly shown in reduced proline-rich proteins, probably due to the loss of biological activity for maintaining oral health. The present study also found a change in TP concentration, in all the Phases, but it is not possible infer which proteins were affected and whether this effect of fluctuation was caused by RT or BC.

Moreover, PX concentration decreased in all Phases of the study and CAT increased after Phase 3 in SWS. These results showed a natural loss of antioxidant capacity of saliva after RT (12) and BC was not capable of increasing these levels. Furthermore, the free radicals and peroxides produced in RT (5) may be responsible for the variation in TP and change in salivary enzyme activity, especially CAT and PX. The amylase concentration also increased, indicating that the BC could stimulate the remnant acinar cells in irradiated patients, mainly the parotid gland cells.

This body of evidence was brought up for discussion with regard to the most opportune time to start BC therapy, concomitantly or after RT. The model of free radicals and peroxides that bind to acinar cell membrane receptors, blocking the activation of the entire intracellular protein signaling cascade in salivary glands (5), could justify the best time for BC therapy. When BC binds to the receptors of the salivary gland cells as a cholinergic agonist, before RT, it could prevent the peroxides and free radicals produced by RT from binding in a way similar to that of a competitive mechanism. Thus, the best protocol for BC therapy seems to be the prophylactic approach, as has been stated in the literature (2,9,10,12,21,23). However, additional studies are necessary to prove this hypothesis and assess other possible changes in saliva composition, salivary flow rate and XT, especially in parotid glands.

Miah *et al.* (26) evaluated the parotid gland function in irradiated patients by the Lashley method, which consists in cups applied over the parotid ducts. Approximately 22% of the patients presented a pre-RT parotid salivary flow rate of 0.08 ml/min unstimulated and 0.1 ml/min stimulated. One year after RT, both unstimulated and stimulated parotid flows were 0.0 ml/min. In addition, some patients reported discomfort during the collection. In this study, a specific collection of parotid gland was not performed due to the flow reduction after RT that would prejudice substantially the sample volume and consequently the biochemical analysis. Most of the patients had completed the RT a long time ago (mean of 21 months) and the hyposalivation was evaluated through UWS and SWS. Moreover, we used this methodology in a previous study (2) and the patients did not complain.

In conclusion, BC therapy used as treatment of XT in patients submitted to head and neck RT, could be important in decreasing the levels of this symptom, but not effective as prophylactic BC therapy. The substantial increase in Amylase concentration suggests that BC stimulates the remnant parotid cells that were not affected by RT.
